# Convulsive Syncope as the Initial Presentation of Massive Postpartum Pulmonary Embolism Leading to Cardiac Arrest: A Case Report

**DOI:** 10.7759/cureus.106883

**Published:** 2026-04-12

**Authors:** Khuloud H Alnuaimi, Asmaa A Shaikh

**Affiliations:** 1 Emergency Department, Sheikh Tahnoon Bin Mohammed Medical City (STMC), Al-Ain, ARE

**Keywords:** cardiac arrest, cesarean section, convulsive syncope, hypoxic-ischemic brain injury, postpartum pulmonary embolism, thrombolysis

## Abstract

Massive pulmonary embolism (PE) remains a leading cause of maternal mortality in the postpartum period, particularly following cesarean delivery. Atypical neurological presentations, including seizure-like activity, may represent convulsive syncope secondary to transient cerebral hypoperfusion rather than true epileptic events, potentially delaying diagnosis and management. We report the case of a 28-year-old multiparous woman (para 5, including two sets of twins) who presented three weeks post-cesarean section with sudden collapse and cardiac arrest. She initially developed abdominal pain followed by brief seizure-like episodes without post-ictal confusion, after which she transiently returned to baseline. During transport, she became unresponsive. On arrival, she was in cardiopulmonary arrest with an estimated downtime of 10 minutes. Advanced cardiac life support was initiated, and sustained return of spontaneous circulation was achieved after approximately one hour of resuscitation. Computed tomography pulmonary angiography demonstrated massive bilateral pulmonary emboli. Systemic thrombolysis with alteplase was administered during resuscitation at a dose of 100 mg over two hours. Despite aggressive critical care management, the patient developed severe hypoxic-ischemic brain injury with persistent myoclonus and ultimately progressed to brain death following a second cardiac arrest. This case highlights the diagnostic challenge of massive PE presenting with atypical neurological features. Seizure-like activity may reflect convulsive syncope due to transient cerebral hypoperfusion and should prompt early consideration of life-threatening cardiovascular etiologies. Heightened clinical suspicion during the high-risk postpartum period remains essential to improve timely diagnosis and outcomes.

## Introduction

Venous thromboembolism (VTE) remains a leading cause of maternal morbidity and mortality worldwide, with pulmonary embolism (PE) representing its most life-threatening manifestation. PE accounts for approximately 9-10% of pregnancy-related deaths and remains a major contributor to preventable maternal mortality, particularly in the postpartum period [1-3]. The incidence of VTE during pregnancy is estimated at 0.5-2.0 per 1,000 pregnancies, reflecting a four- to five-fold increased risk compared with nonpregnant women [1,2].

The increased risk of VTE in pregnancy is driven by physiological changes consistent with Virchow’s triad, including hypercoagulability, venous stasis, and endothelial injury [3,4]. This risk peaks during the postpartum period, particularly within the first two to six weeks after delivery, during which it may increase up to 80-fold compared with nonpregnant women [2-4]. Cesarean delivery is an independent and significant risk factor, conferring approximately a fourfold increased risk compared with vaginal delivery [4,5]. Additional contributors include multiparity, multiple gestation, assisted reproductive technology, and reduced mobility [3-5].

The clinical presentation of PE is often nonspecific and variable, frequently leading to diagnostic delays. While dyspnea, chest pain, and syncope are the most common presenting symptoms, atypical manifestations may occur [6]. Neurological presentations, including seizure-like activity or convulsive syncope, are rare but clinically significant and are thought to result from transient cerebral hypoperfusion secondary to acute right ventricular failure and reduced cardiac output [7-9].

Massive PE is defined by hemodynamic instability and carries a high mortality rate, particularly when complicated by cardiac arrest [10,11]. Despite advances in diagnostic strategies and therapeutic interventions, outcomes remain poor when diagnosis is delayed or when prolonged hypoperfusion occurs.

We report a rare and catastrophic case of massive postpartum PE presenting initially with seizure-like activity followed by cardiac arrest, highlighting the diagnostic challenges of atypical neurological presentations and emphasizing the importance of early recognition and timely intervention in high-risk postpartum patients.

## Case presentation

A 28-year-old postpartum woman, three weeks following cesarean section for twin delivery, presented with sudden collapse and cardiac arrest. She had a background of multiparity (para 5), multiple in vitro fertilization pregnancies, and prior gastric sleeve surgery. Her antenatal course was otherwise unremarkable.

On the day of presentation, she developed acute abdominal pain, followed within minutes by two brief episodes of seizure-like activity without post-ictal confusion. She returned transiently to baseline before becoming unresponsive shortly thereafter during transport.

On arrival, she was in cardiopulmonary arrest with an estimated downtime of approximately 10 minutes, with no reported bystander cardiopulmonary resuscitation before arrival. Advanced Cardiac Life Support (ACLS) was initiated immediately. The initial rhythm was asystole. The patient required multiple cycles of high-quality cardiopulmonary resuscitation with intravenous epinephrine, airway control with endotracheal intubation, and vasopressor support. Return of spontaneous circulation (ROSC) was achieved intermittently at 10:08, 10:34, and 10:57, with sustained ROSC after approximately one hour of resuscitation.

During resuscitation, a structured evaluation for reversible causes was performed using the H’s and T’s approach. PE was considered under “thrombosis” based on the patient’s high-risk postpartum status following recent cesarean delivery, preceding acute abdominal pain, and sudden collapse with seizure-like activity suggestive of convulsive syncope, in the absence of an alternative cause.

Severe metabolic acidosis was identified, characterized by a pH of 7.01 and low bicarbonate (11 mmol/L), accompanied by markedly elevated lactate levels and hyperkalemia (up to 6.8 mmol/L). These abnormalities were promptly recognized and corrected as part of ongoing resuscitative efforts (Table 1). The laboratory findings were consistent with severe high-anion gap metabolic acidosis, reflecting profound tissue hypoperfusion and impaired oxygen delivery, raising concern for an underlying obstructive shock state, such as massive PE.

**Table 1:** Laboratory results on presentation. Base excess reflects the metabolic component of acid-base balance; negative values indicate metabolic acidosis.

Parameter	Result	Reference range
pH (arterial blood gas)	7.01	7.35–7.45
Bicarbonate (HCO₃⁻)	11 mmol/L	22–28 mmol/L
Base excess	−19 mmol/L	−2 to +2 mmol/L
Lactate	12.5 mmol/L	0.5–2.0 mmol/L
Potassium (K⁺)	6.8 mmol/L	3.5–5.0 mmol/L
D-dimer	>35,200 µg/L	<500 µg/L

Despite correction of metabolic derangements, the patient remained hemodynamically unstable, raising suspicion for an obstructive etiology, particularly PE.

**Figure 1:**
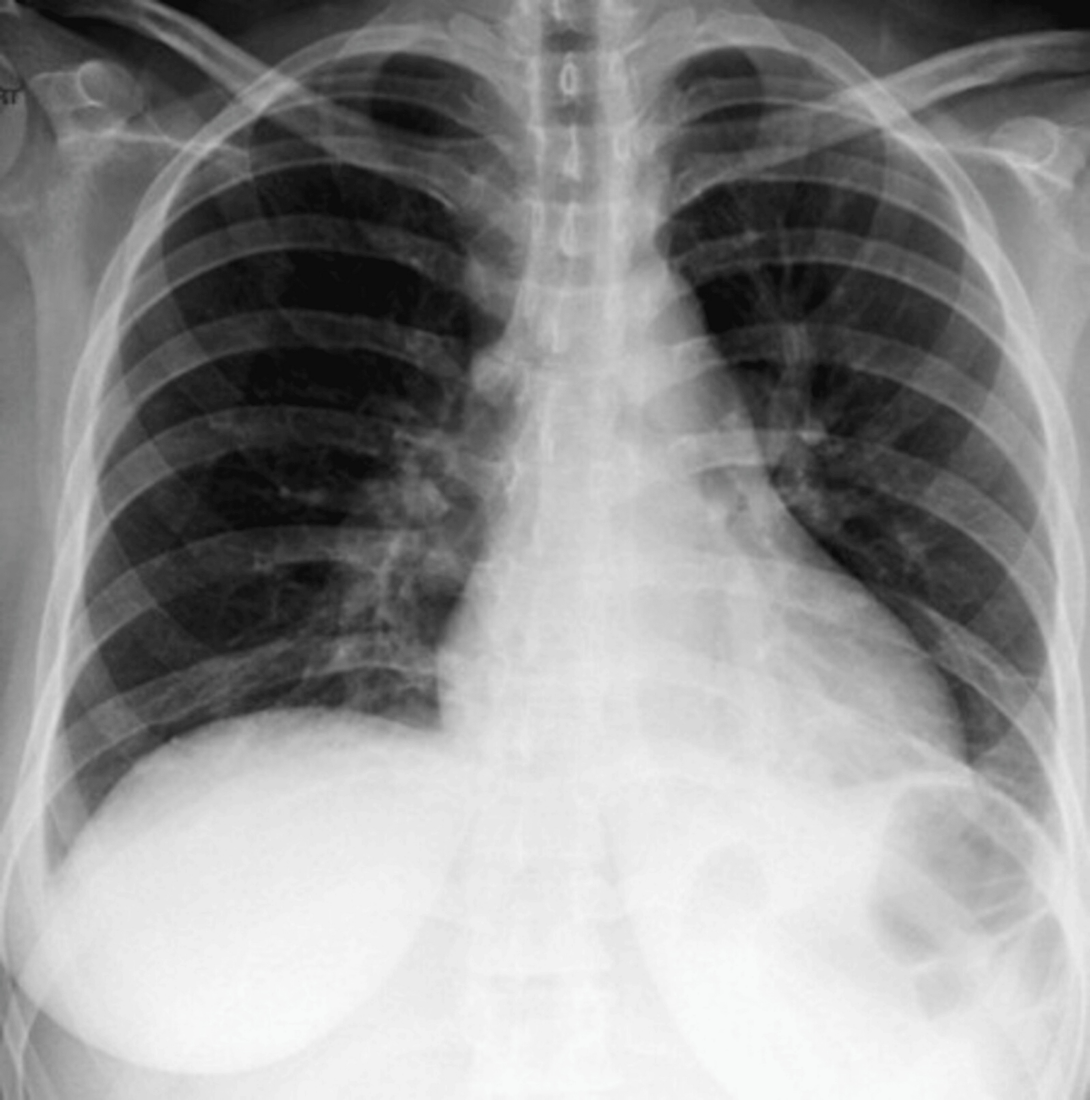
Portable chest radiograph demonstrating no acute cardiopulmonary abnormality, with no focal consolidation or pneumothorax.

A portable chest radiograph showed no acute cardiopulmonary abnormality; however, a normal chest X-ray does not exclude PE and has limited sensitivity in this context (Figure 1).

Given the postpartum status and preceding neurological symptoms, differential diagnoses included eclampsia, amniotic fluid embolism, intracranial pathology, and PE. Empirical magnesium sulfate was administered.

**Figure 2:**
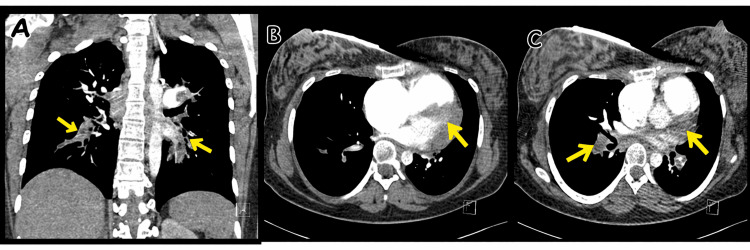
Computed tomography pulmonary angiography demonstrating extensive bilateral pulmonary embolism with central and segmental filling defects (arrows), consistent with a high clot burden.

Urgent computed tomography (CT) pulmonary angiography demonstrated large filling defects involving both the right and left main pulmonary arteries with extension into segmental branches, consistent with a high clot burden (Figure 2).

**Figure 3:**
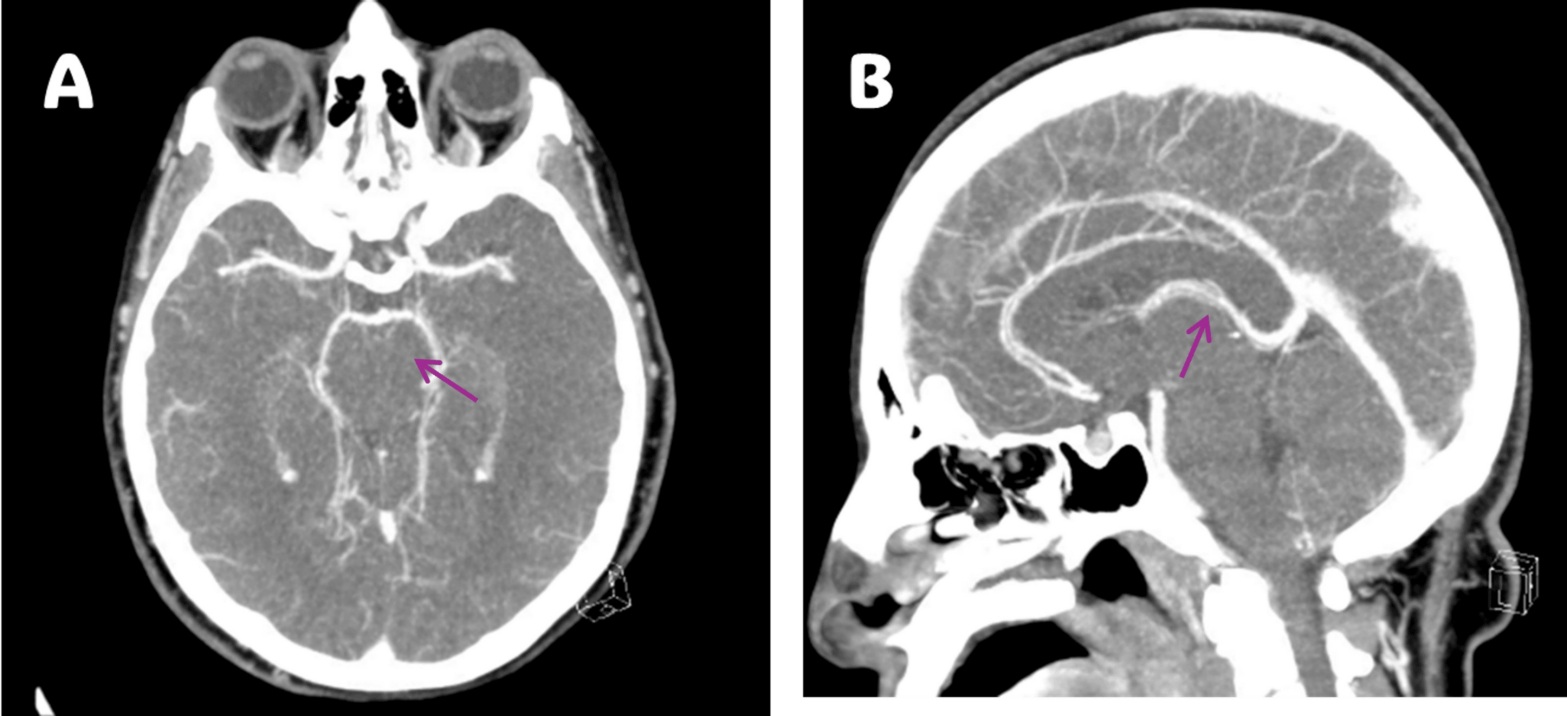
Computed tomography (CT) brain images. (A) Axial CT brain and (B) sagittal CT brain, both demonstrating no intracranial hemorrhage or venous sinus thrombosis, with findings suggestive of early diffuse cerebral edema (arrows).

CT brain and cerebral venography demonstrated no acute intracranial hemorrhage, large territorial infarction, or venous sinus thrombosis, with findings suggestive of early diffuse cerebral edema (Figure 3).

Systemic thrombolysis with alteplase was initiated promptly following diagnostic confirmation of PE. The patient was subsequently transferred to the intensive care unit (ICU) for ongoing management.

Transthoracic echocardiography demonstrated preserved left ventricular function with no significant valvular abnormalities and only mild pulmonary hypertension. During her ICU course, she developed persistent myoclonic activity. Electroencephalography revealed diffuse slowing without epileptiform activity, consistent with global cerebral dysfunction. Magnetic resonance imaging of the brain confirmed hypoxic-ischemic injury.

Despite transient stabilization, she developed a second cardiac arrest on day four with rapid ROSC. Repeat imaging demonstrated diffuse cerebral edema with herniation. She subsequently developed central diabetes insipidus. Formal neurological assessment confirmed brain death, followed by cardiac death.

The sequence of clinical events from initial presentation through resuscitation, diagnosis, and outcome is summarized in Table 2.

**Table 2:** Clinical timeline of events. ED = emergency department; ACLS = advanced cardiovascular life support; ROSC = return of spontaneous circulation; CXR = chest X-ray; CT = computed tomography; PE = pulmonary embolism; ICU = intensive care unit; EEG = electroencephalogram; MRI = magnetic resonance imaging

Time	Event
Pre-hospital	Abdominal pain → two brief seizure-like episodes
Transport	Sudden unresponsiveness
ED arrival	Cardiac arrest (asystole), ACLS initiated
10:08	First ROSC achieved
10:12	Re-arrest
10:34	Second ROSC
10:51	Re-arrest
10:57	Sustained ROSC
Early ED	Severe acidosis (pH 7.01), lactate >12 mmol/L
Initial imaging	CXR: nonspecific
Post-ROSC	CT pulmonary angiography → massive bilateral PE
Same day	Alteplase administered
ICU course	Myoclonic jerks, EEG: diffuse slowing
MRI brain	Hypoxic-ischemic injury
Day 4	Second cardiac arrest
Post-event	Cerebral edema + herniation
Final outcome	Brain death → cardiac death

## Discussion

PE remains a major cause of maternal morbidity and mortality, particularly in the early postpartum period, where the risk of VTE is markedly elevated [1-3]. This patient presented three weeks following cesarean delivery, placing her within the highest-risk window. The presence of multiple risk factors, including cesarean delivery, multiparity, twin pregnancy, and assisted reproductive technology, likely contributed synergistically to thrombus formation [3-5].

Pregnancy induces a hypercoagulable state characterized by increased levels of procoagulant factors, reduced fibrinolysis, and venous stasis, collectively fulfilling Virchow’s triad [3,4]. These physiological adaptations, although protective against hemorrhage, significantly increase susceptibility to thromboembolic events, particularly when compounded by surgical delivery and relative immobility.

The clinical presentation of PE is frequently nonspecific and may be atypical, contributing to delayed recognition. Neurological manifestations such as seizure-like activity are uncommon but clinically important presentations. These episodes are increasingly recognized as convulsive syncope, resulting from transient cerebral hypoperfusion secondary to acute right ventricular failure and reduced cardiac output [7-9]. In this case, the absence of post-ictal confusion and rapid return to baseline supports convulsive syncope rather than primary seizure activity. Additional distinguishing features include the typically brief duration, identifiable trigger (such as acute hemodynamic compromise), and rapid recovery without a prolonged post-ictal state, in contrast to epileptic seizures. Notably, syncope in PE has been associated with increased rates of hemodynamic instability and right ventricular dysfunction, which correlate with worse clinical outcomes [12].

In this case, the initial chest radiograph was unremarkable, emphasizing the limited sensitivity of chest X-ray in PE and the need for a high index of suspicion despite nonspecific or normal initial imaging. This underscores the limitation of relying on initial imaging in isolation and reinforces the importance of clinical judgment in high-risk patients.

Cardiac arrest secondary to PE represents a form of obstructive shock and is associated with high mortality. PE is estimated to account for approximately 2-5% of cardiac arrest cases, although this is likely underestimated [10]. The systematic application of ACLS, particularly the identification of reversible causes through the H’s and T’s framework, is critical in such scenarios. Recognition of PE under “thrombosis” is essential to guide timely diagnostic and therapeutic interventions [13,14].

Systemic thrombolysis remains the cornerstone of treatment in high-risk PE, including cases complicated by cardiac arrest. Evidence suggests that thrombolysis is associated with improved rates of ROSC and short-term survival in patients with confirmed PE [14,15]. However, outcomes remain highly dependent on the duration of hypoperfusion. In this case, despite prompt thrombolysis, prolonged resuscitation resulted in severe hypoxic-ischemic brain injury, underscoring the critical importance of early diagnosis. Targeted temperature management and other neuroprotective strategies were considered; however, the extent of neurological injury following prolonged downtime limited their potential benefit. This case highlights the importance of maintaining a high index of suspicion for PE in postpartum patients presenting with atypical features, to facilitate early diagnosis and timely intervention.

Diagnosis of PE in pregnancy is inherently challenging due to overlapping physiological symptoms and limitations of conventional biomarkers. Clinical decision tools such as the pregnancy-adapted YEARS algorithm have demonstrated safety in reducing unnecessary imaging in stable patients [16]. However, in this case, the patient presented with hemodynamic instability and cardiac arrest, precluding the use of structured diagnostic algorithms and necessitating immediate imaging and empiric treatment without delay.

This case emphasizes several important clinical implications. First, PE should be considered in postpartum patients presenting with unexplained neurological symptoms or sudden collapse, even in the absence of typical cardiopulmonary features. Second, seizure-like activity may represent convulsive syncope rather than a primary neurological disorder. Third, early integration of PE into the ACLS framework as a reversible cause of cardiac arrest is essential for timely intervention. Finally, systematic risk assessment and appropriate thromboprophylaxis remain critical to reducing preventable maternal mortality associated with postpartum PE [1,3,5].

## Conclusions

This case underscores the catastrophic potential of massive PE in the postpartum period and the diagnostic challenges associated with atypical neurological presentations. Seizure-like activity may represent convulsive syncope due to acute cerebral hypoperfusion and should prompt early consideration of life-threatening cardiovascular causes. Early integration of PE into the ACLS framework, particularly as a reversible cause of cardiac arrest, is essential for timely diagnosis and intervention. Notably, this case highlights a rare presentation of massive postpartum PE initially manifesting as seizure-like activity progressing rapidly to cardiac arrest, emphasizing the diagnostic difficulty in the absence of typical cardiopulmonary symptoms. Despite appropriate thrombolysis, prolonged cardiac arrest may result in irreversible neurological injury, underscoring that early recognition and prevention during the high-risk postpartum period remain critical to reducing maternal mortality.
